# Direct current induced short-term modulation of the left dorsolateral prefrontal cortex while learning auditory presented nouns

**DOI:** 10.1186/1744-9081-5-29

**Published:** 2009-07-15

**Authors:** Stefan Elmer, Marcel Burkard, Basil Renz, Martin Meyer, Lutz Jancke

**Affiliations:** 1 Department of Neuropsychology, University of Zurich, Switzerland

## Abstract

**Background:**

Little is known about the contribution of transcranial direct current stimulation (tDCS) to the exploration of memory functions. The aim of the present study was to examine the behavioural effects of right or left-hemisphere frontal direct current delivery while committing to memory auditory presented nouns on short-term learning and subsequent long-term retrieval.

**Methods:**

Twenty subjects, divided into two groups, performed an episodic verbal memory task during anodal, cathodal and sham current application on the right or left dorsolateral prefrontal cortex (DLPFC).

**Results:**

Our results imply that only cathodal tDCS elicits behavioural effects on verbal memory performance. In particular, left-sided application of cathodal tDCS impaired short-term verbal learning when compared to the baseline. We did not observe tDCS effects on long-term retrieval.

**Conclusion:**

Our results imply that the left DLPFC is a crucial area involved in short-term verbal learning mechanisms. However, we found further support that direct current delivery with an intensity of 1.5 mA to the DLPFC during short-term learning does not disrupt longer lasting consolidation processes that are mainly known to be related to mesial temporal lobe areas. In the present study, we have shown that the tDCS technique has the potential to modulate short-term verbal learning mechanism.

## Background

Memory is a key issue in cognitive neuroscience and probably constitutes one of the most complex cognitive functions. The human memory system comprises various memory subtypes controlled by complex cortico-subcortical networks [1]. The prefrontal cortex (PFC) is a core structure within these networks and plays an essential role in the integration of information and the management of multiple tasks [2]. Indeed, the PFC is crucial in subserving higher cognitive functions like memory, planning, goal-oriented behaviour, role learning, attention and inhibition.

The advent of functional neuroimaging techniques has brought with it an accumulation of evidence pointing to the involvement of the prefrontal cortex in the encoding and retrieval of verbal and non-verbal stimuli [2-4], and in the control of working memory processes [5,6]. There is also some evidence for functional asymmetries of the PFC during encoding and retrieval of verbal or nonverbal material. Several authors [7,8] have emphasised that functional hemispheric dominance in memory tasks is contingent on the memory subprocesses involved and on the verbalisability of the stimuli, thus verbal stimuli recruiting more strongly left sided neural networks. While there is no doubt that the PFC is involved in learning processes, it is unclear as to whether and how strongly the PFC is engaged in controlling long-term memory processes. In this context, different lesion studies have not consistently shown memory impairments with frontal lesions [9].

We applied transcranial direct current stimulation (tDCS) with the aim of examining hemispheric dominance during an auditory verbal memory task. The decision to modulate the right and left PFC separately was motivated by the hemispheric encoding/retrieval asymmetry (HERA) model, which is a process-specific description of experimental data provided by a large set of functional neuroimaging studies [10]. The tDCS technique enables the investigation of the role of particular brain areas in controlling various cognitive tasks by modulating the degree of cortical excitability with a weak electrical current in the form of direct current brain polarization [11,12]. Depending on the polarity of the applied current, neural firing rates increase (anodal) or decrease (cathodal), this being probably due to an induced change in resting membrane potentials [13,14]. The efficacy of tDCS to induce acute modifications of membrane polarity depends on current delivery which determines the induced electrical field strength, this being the quotient of current strength and electrode size [15]. Data from animal studies suggest that direct current-induced changes in neuronal excitability persist beyond the period of stimulation when tDCS is applied for more than about 3 minutes and that it remains stable for at least 1 hour when delivered for longer than 10 minutes [14]. Electrophysiological findings [16], neuroimaging studies [17,18], and neural computation modelling [19] convincingly delineate the physiological effect of direct current application on the human brain.

Only a paucity of the tDCS studies to date has explored the modulation of prefrontal areas during explicit memory tasks and to our knowledge, none of these used auditory presented nouns as stimuli. For example, a previous research that evaluated the effect of tDCS on a visual letter working memory task reported that anodal stimulation of the left DLPFC increased performance accuracy when compared with sham stimulation (baseline) on the same side [20]. Another study investigated consolidation of declarative memories [21] and found that bilateral anodal direct current stimulation at frontocortical electrode sites affected declarative memory when applied during sleep. Further evidence for the effect of direct current stimulation on memory functions in healthy humans arises from the same group. Marshall et al. [22] investigated the influence of direct current on a visual letter working memory task applying bilateral electrodes at fronto-lateral locations. The authors reported slowed reaction time during both anodal and cathodal stimulation, this suggesting that any kind of electrical stimulation hampers neuronal processes related to response selection and preparation. Otherwise, further research has evidenced facilitation of learning and memory processes by tDCS application to the prefrontal cortex [13,23].

To our knowledge, none of the published studies presented auditory verbal stimuli during tDCS application in order to test its modulatory effect on both short-term learning and subsequent long-term retrieval. We therefore sought to use the tDCS method to examine the question of relative hemispheric specialisation of the DLPFC in auditory verbal learning mechanism. We hypothesised in view of the findings of some neuroimaging studies [7,8] verbal learning should mainly be modulated by stimulation of the left prefrontal cortex. Secondly, based on a prior electrophysiological study with verbal material [20], we expected a better learning performance during anodal stimulation of the left DLPFC, and we assumed a decrease in performance during cathodal stimulation of the same hemisphere. Finally, we sought to find that DLPFC modulation during short-term learning will also influence long-term retrieval.

## Methods

### Subjects

Twenty male volunteers (native Swiss-German) ranging in age from 19 to 26 years (mean age 22.3, SD 2.3) were recruited for the experiment. All participants were university students with a similar level of education (high school degree, mean years of education in school 14.45, SD 1.73). According to the Annett-Handedness-Questionnaire [24] all subjects were consistently right-handed, gave written consent in accordance with procedures approved by the local ethics committee (ethic committee of the canton of Zürich, specialized subcommittee for psychiatry, neurology and neurosurgery, Oetwil am See, Switzerland) and were paid for participation.

### Procedure and stimuli

The participants were placed in a comfortable chair in front of a screen and two loudspeakers positioned at an angle of about 90 degrees in the horizontal plane and performed the experiment in a well-lit and quiet room. Volunteers were assigned to one of two groups each performing the same three stimulation blocks (anodal, cathodal and sham) in a randomised order. During the experiment, the participants fixated a small cross in the middle of the screen while single nouns were presented (loudness ~50 dB sound pressure level).

The auditory stimuli consisted of 25 German nouns out of the VLMT test (see Table 1) recorded from a native German speaker and processed with an audio-software (MAGIX Audio Studio 03 deLuxe, Magix AG, Berlin, Germany). All stimuli were normalized for amplitudes and re-checked by means of the PRAAT speech editing software [25]. Auditory stimuli presentation was controlled by "presentation" software (Neurobehavioral Systems, USA, Version 0.70) [26]. The nouns were presented every 2 seconds, word duration and ISI were about 1 second.

**Table 1:** Auditory stimuli

**Form A**	**Form C**	**Form D**
Trommel	Geige	Horn
Vorhang	Fenster	Tür
*Hummel*	*Biene*	*Fliege*
Glocke	Lampe	Seil
*Lunge*	*Darm*	*Niere*
Kaffee	Museum	Gericht
Schule	Tee	Kakao
*Zimt*	*Paprika*	*Salz*
Eltern	Reise	Wagen
*Haar*	*Kamm*	*Bürste*
Mond	Sonne	Sterne
Garten	Wiese	Baum
*Afrika*	*Australien*	*Amerika*
Hut	Treppe	Mantel
*Ananas*	*Gurke*	*Traube*
Pfarrer	Maurer	Bauer
Nase	Zunge	Mund
*Linde*	*Ahorn*	*Buche*
Truthahn	Tiger	Gans
*Kegeln*	*Hockey*	*Karate*
Farbe	Musik	Form
Haus	Stadt	Land
*Bügel*	*Hebel*	*Arm*
Fluss	See	Regen
*Säge*	*Nagel*	*Schraube*

The nouns printed in regular font are those of the original version of the VLMT test (form A, B & C). We expanded the original list in order to avoid ceiling effects (italic printed nouns).

To assess short-term learning and long-term retrieval, three parallel forms of the VLMT test (Verbaler Lern- und Merkfähigkeitstest, i.e. verbal learning test, form A, C and D) were presented in randomized order across subjects and groups. In order to avoid ceiling effects, the original version of 15 semantically unrelated nouns was expanded to 25, controlling for word frequency (on the basis of a comprehensive search using the "google" search system) [27] and categories. The distractor lists of the original version were not applied and the participants performed only three instead of the five encoding runs of the original version. Every participant had 120 seconds after each encoding run for the immediate retrieval of the heard nouns. The participants had to speak the remembered nouns into a microphone and all responses were recorded. The total number of correctly remembered words after the third run was taken as an objective measure of short-term learning achievement [28]. In accordance with the original VLMT test, late retrieval was tested about 25 minutes after the first encoding trial. The retrieval score was based on the number of correctly remembered words after the delay period [28].

### Experimental schedule

**Figure 1:**
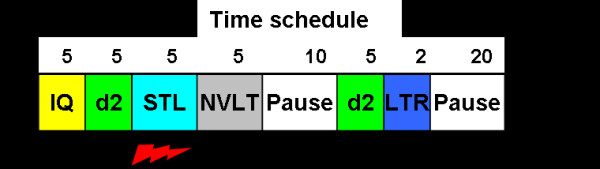
**Schedule of the first block**. IQ = MWT A intelligence test; d2 = short-term attention test; STL = short-term learning test; NVLT = non verbal learning test; LTR = long–term retrieval. The red flash symbolizes the time-frame of direct current delivery.

Prior to each session, the subjects performed a German verbal intelligence (MWT A) and a short-term attention test (d2) with the intention of controlling for group homogeneity in task-relevant cognitive abilities. Each block (in total 3, only differing in current application) comprised the following trial sequence: (I) "VLMT short-term learning test (STL)", (II) "NVLT non-verbal learning test", (III) "Pause", (IV) "d2 attention test", (V) "VLMT long-term retrieval test (LTR)" and (VI) "Pause". During trial (I), the participants had to encode and immediately retrieve the auditory presented words of the VLMT test three times. Simultaneous sham, anodal or cathodal stimulation was delivered via a frontolateral electrode. Trial (II) was a nonverbal recognition test with the intention of avoiding active memory strategies until later retrieval (V). After a short pause (III) in which a silent cartoon was presented, a second d2 test followed (IV). The pause had the function of excluding after-effects of current delivery on cortical excitability/suppression before the later retrieval (LTR) was next (V). The d2 test was inserted to control attention during the entire experiment. Before starting the next block the participants had a second pause (20 minutes) (VI) designed to distract the participants by means of a silent cartoon before the next parallel form of the memory test was presented. Each of the three blocks had duration of 47 minutes. Thus the total duration of the experiment (including both pre-experimental d2 and MWT tests) was about 130 minutes. Figure 1 indicates the schedule of one block.

### Transcranial direct current stimulation (tDCS)

The experiment was conducted with a transcranial direct current stimulator. Current was transferred by a saline-soaked pair of surface sponge electrodes and delivered by a battery-driven constant current stimulator (eldith, neuroConn GmbH, Germany). The electrodes were applied unilaterally (i.e. to the right or left hemisphere) at fronto-lateral location (F3 or F4 according to the international 10/20 system) and over the mastoid. This method of DLPFC localisation has been used in previous studies [11,20-22] and been confirmed as an appropriate method of localisation by neuronavigation techniques [29]. The fronto-lateral electrodes we used had an area of 28 cm^2 ^(7 cm × 4 cm). We choose a mastoid electrode with a larger surface (100 cm^2^, 10 cm × 10 cm) in order to reduce current density at the posterior-lateral brain side. Cathodal and anodal stimulation were delivered with a constant current of 1.5 mA. The baseline condition (sham) was performed without any tDCS influence. Stimulation was applied for a period of 5 minutes, with a linear fade in/fade out of 10 seconds and was congruent with the duration of the three encoding trials (see VLMT test). Anodal/cathodal/sham application was randomly controlled across subjects and groups. Both groups run through the same experimental setting but differed in stimulation side (right or left sided sham/cathodal/anodal current application).

### Psychometric tests

The MWT (Mehrfachwahl-Wortschatz-Intelligenz-Test) is a clinical test for assessing the verbal intelligence quotient. The entire test can be executed in about 300 seconds and allows fast screening of general verbal intellectual capacities.

Each d2 short-term attention test had duration of 280 seconds. Score evaluation was based on the difference between the sum of correctly arranged items and the confusion errors [30]. During the entire experiment, subjects performed a total of 4 d2 tests (see Figure 1). In order to avoid redundancy, the original version was scrambled, forming 4 parallel versions.

The applied NVLT test (nonverbaler Lerntest, i.e. non-verbal learning test) comprised 120 meaningless figures. Each figure was presented visually on the screen for 3 seconds. Eight figures were presented 5 times. The subject had to indicate by pressing a keyboard button whether the figures had been presented before or not. The performance scores were not further analysed because they were beyond the main interest of this study.

### Control variables

For the purpose of further data analysis it is important that both groups were comparable in the following task-relevant variables: age, years of education, verbal intelligence and short-term attention. It was also relevant that both groups showed a similar level of achievement during sham stimulation and that attention was comparable between both groups during the entire experiment. To control for the influence of these variables, we statistically compared the two groups.

## Results

### Control variables

Before subjecting age, years of education, intelligence/attention scores and VLMT performance during sham stimulation across both groups to parametrical statistical testing, we ascertained that data were normally distributed (Kolmogorov-Smirnov-test). T-tests for independent samples did not reveal significant differences in these control variables among groups. In addition we computed d2 scores in a 2 × 3 repeated-measure ANOVA looking for attention effects across groups among the three blocks. We tested the prerequisites for an analysis of variance, namely homogeneity of variances (Mauchly's test of sphericity) and normal data distribution (Kolmogorov-Smirnov-test). Neither the main effects "stimulation mode" (SM) and "group" (G) nor the interaction "stimulation mode" × "group" (SMG) reached significance. Thus we assumed a comparable attention level in both groups.

### Short-term learning

Short-term learning was quantified by evaluating the total number of remembered words after the third encoding run (see Figure 1). For this purpose, we computed a repeated-measure 3 × 2 ANOVA with the following independent variables: SM (sham/anodal/cathodal) and G (RHG and LHG). The ANOVA revealed no significant main effects but a significant SM × G interaction (SMG: F(1,18) = 7.2, p = .015, eta^2 ^= .72).

**Figure 2:**
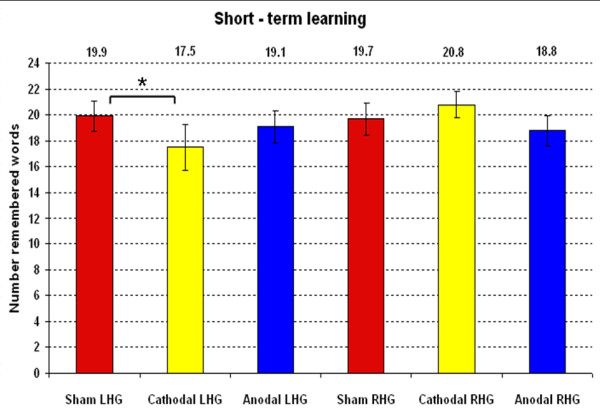
**Short-term learning performance**. Mean values and standard errors of short-term learning performance during every stimulation mode for both hemispheres. * depicts significance, p < .05.

To further examine this interaction we computed two separate one-way ANOVAs, one for each group (RHG/LHG, repeated-measure). This statistical analysis was applied to examine the significant interaction we found in the higher level 3 × 2 ANOVA. The outcome of this procedure revealed a significant SM effect in the left but not in the right hemisphere group (LHG: F(1,9) = 6.0, p = .037, eta^2 ^= .59), thus evidencing that tDCS application had a significant effect only in the LHG. To further elucidate the SM effect found in the LHG, we computed three t-tests (one-tailed) for dependent samples (sham vs. anodal/sham vs. cathodal/anodal vs. cathodal). The results of these post-hoc comparisons showed a significant result only for the Sham vs. Cathodal contrast (sham vs. cathodal: t(9) = 2.44, p = .018, one tailed; Bonferroni corrected p value = .016). Figure 2 and Table 2 show the significant results of the post-hoc analysis.

**Table 2:** Post-hoc comparisons

	**one-way ANOVAs**	
**Group**	**F-value**	**p-value**
LHG	6.00	0.037
		
	**t-tests**	
**Contrast**	**t-value**	**p-value** **(one-tailed)**

Sham vs. Cathodal	2.44	0.018

Significant results of the post-hoc analyses.

### Long-term retrieval

We recorded the correctly remembered words after the delay period as the index for long-term retrieval. We computed a repeated-measure 3 × 2 ANOVA with the factors SM and G. The analysis revealed no significant main (SM, G) or interaction effects.

## Discussion

The aim of this study was to examine the behavioural effects of right and left-hemisphere frontal direct current delivery while memorizing auditory presented words on short-term learning and subsequent long-term retrieval. Our results provide evidence for a short-term effect that appeared not to influence consolidation mechanisms. As a main result, we found that left-side cathodal tDCS application induced poorer performance than sham tDCS application in the same area. Our results demonstrate that the left DLPFC is a crucial area involved in short-term verbal learning mechanisms and that tDCS is a suitable method that permits to modulate verbal memory functions. In line with this, several functional imaging studies consistently showed prefrontal activation during committing to memory various types of stimuli [31] but the issue of lateralization has been shown to depend on the material presented [7,8] as well as on specific memory processes within the classical framework [32]. Our results corroborate findings of various neuroimaging studies [33,34] and confirm the relevance of the left prefrontal area regarding learning processes of auditory presented verbal contents.

Only few tDCS studies to date have focussed on memory functions, and none of these used auditory presented verbal stimuli. Previous tDCS studies mainly collected behavioural data by performing verbal or non-verbal working memory tasks, disregarding other subtypes of memory functions. For example, by using visually presented verbal stimuli Fregni et al. [20] and also Marshall et al. [22] tested the possibility of influencing frontal-lateral brain areas performing verbal working memory tasks. Both studies produced controversial results. Marshall et al. applied bilateral electrodes on the DLPFC and reported slowed reaction times during both anodal and cathodal stimulation compared with sham. Fregni et al. reported that anodal stimulation of the left PFC lead to an increased performance compared with sham stimulation. In contrast, our results using auditory stimuli show that cathodal but not anodal stimulation of the left hemisphere significantly alters short-term learning performance. Therefore, our results lead us to suggest that cathodal stimulation over the left DLPFC provokes a direct or indirect down-regulation of brain areas involved in short-term auditory verbal learning mechanisms. Furthermore, our results are congruent with a recently published study [35] that demonstrated the potential of cathodal direct current stimulation to modulate the functional contribution of posterior-lateral brain areas for tone memory processes.

Our results suggest a pattern of material specific activation principally involving the left language-dominant hemisphere. Somewhat deviating from the HERA model [10], our results suggest that task-related activation was lateralized primarily according to the nature of the material (verbal) rather than the stage of episodic operations involved (encoding or retrieval). In line with this, Wagner et al. [8] revealed a pattern of material-specific left-sided prefrontal activation that was similar during episodic encoding as well as retrieval of visual presented verbal contents using fMRI. In a further fMRI investigation Lidaka et al. [34] demonstrated a strong relationship between retrieval success for words and activation in the left prefrontal cortex. The results of these two studies are also consistent with neuropsychological evidence that left and right frontal lesions differentially impact verbal and non-verbal episodic memory [36], such that left frontal lesions more strongly impair verbal episodic memory functions. In general, our findings replicate previous reports on the functional material-specific asymmetry of prefrontal activation during verbal episodic memory tasks [8,34,37].

Finally, our data suggest that prefrontal direct current delivery did not affect the memory consolidation mechanism mainly known to be related to mesial temporal areas [38,39]. If the consolidation mechanism were disturbed by means of the application of frontal tDCS protocols, then we should have observed a better performance during long-term retrieval after sham than after cathodal stimulation of the left hemisphere. Therefore, it is plausible to conclude that the weak current as applied in this study did not modulate mesial temporal regions involved in consolidation processes. Otherwise, the memory performance data depicted in Table 3 leads us to suggest that the null effect we found during the long-term retrieval condition is probably due to higher forgetting rates in the LHG during the sham condition. The reason for this trend is entirely unclear and any kind of explanation is speculative and therefore does not merit further attention. Our paradigm does not permit any further insight into this effect. Subsequent studies may be able to pursue this issue more closely.

**Table 3:** Memory scores & forgetting rates

	**Sham RHG**	**Sham LHG**	**Anodal RHG**	**Anodal LHG**	**Cathodal RHG**	**Cathodal LHG**
**STL**	19.7	19.9	18.8	19.1	20.8	17.5
**LTR**	18.3	17.8	17	17.3	17.7	15.9
**Δ**	1.4	**2.1**	1.8	1.8	3.1	**1.6**

Mean memory scores during STL and LTR. Δ shows the mean forgetting rates.

## Limitations

A methodological limitation of tDCS protocols is the low spatial resolution and the fact that the modulation of a particular brain area's response to a certain stimulation reflects a limited view of a large-scale functional network [40]. Consequently, the tDCS method implies that a distinct brain region is involved in computational processes that are in fact part of a more complex system [41].

## Conclusion

The aim of the present study was to examine hemispheric dominance while learning auditory presented nouns. We designed a study in which the participants memorized auditory presented verbal stimuli while direct current stimulation was delivered to the DLPFC with a view to examining the modulatory impact of this on short-term learning and long-term retrieval. We examined the behavioural effects of both left and right-side stimulation to gain more knowledge about the distinct or overlapping neural networks involved in learning verbal stimuli. To our knowledge, none of the studies that have applied tDCS to address issues in memory research have presented auditory verbal stimuli to test the effects of direct current on both short-term learning and subsequent long-term retrieval.

Our results indicate that only cathodal tDCS elicits short-term behavioural effects on verbal memory performance. In particular, left-sided stimulation impaired memory performance compared with sham tDCS. The present study demonstrates that the left DLPFC plays a pivotal role while learning auditory presented verbal stimuli. It is remarkable that a complex cognitive function such as verbal memory can be modulated by external stimulation of the brain.

The tDCS technique has a great potential for future applications. Due to the ease of utilisation, the tDCS method enables the testing of hypotheses on memory functions that emerging from basic neuroscience studies and neuroimaging protocols in humans with and without brain lesions [13]. Furthermore, the tDCS application could be a fruitful approach for the treatment of pathologies affecting memory functions.

## Competing interests

The authors declare that they have no competing interests.

## Authors' contributions

SE designed the experimental paradigm, performed the statistical analysis and drafted the manuscript. MB contributed to the hypothesis, to the design and performed the data acquisition. BR contributed to the hypothesis, the design and performed the data acquisition. MM participated in the design/coordination of the study and contributed to the manuscript. LJ conceived of the study, contributed to the hypothesis, design, results, discussion and to the preparation of the manuscript. All authors read and approved the final manuscript.
